# 

**Published:** 1899-09

**Authors:** 


SEPTEMBER, 1899.

*Vol. XVII.* *No.* **65.**

LIBRARY
Indexed
S. G. O.

### THE BRISTOL MEDICO-CHIRURGICAL JOURNAL

**A Quarterly Journal of the Medical Sciences for the West of England and South Wales**

*PUBLISHED UNDER THE AUSPICES OF*

*THE BRISTOL MEDICO-CHIRURGICAL SOCIETY.*

LIBRARY
SURGEON GENERAL'S OFFICE
OCT. 30 - 1899

"Scire est nescire, nisi id me
Scire alius sciret."

*Editor:* R. SHINGLETON SMITH, M.D., B.Sc.

WITH WHOM ARE ASSOCIATED

J. MICHELL CLARKE, M.A., M.D., W. H. HARSANT,
B. M. H. ROGERS, B.A., M.D., B.Ch., AND JAMES SWAIN, M.S., M.D.

*Assistant-Editor:* L. M. GRIFFITHS.

*Editorial Secretary:* JAMES TAYLOR.

BRISTOL: J. W. ARROWSMITH.
London: J. & A. Churchill, 7 Great Marlborough Street.

